# Secular trends in body weight perception among Norwegian adolescents: a 28-year cross-sectional analysis (1994–2022)

**DOI:** 10.1038/s41598-025-00439-y

**Published:** 2025-05-15

**Authors:** Catharina Wold Robson, Anne-Siri Fismen, Jens Christoffer Skogen, Ellen Merethe Melingen Haug

**Affiliations:** 1 Department of Health Promotion and Development, University of Bergen, Muséplassen 1, Bergen, 5007 Norway; 2 Department of Health and Caring Sciences, Western Norway University of Applied Sciences, Inndalsveien 28, Bergen, 5063 Norway; 3 Department of Health Promotion, Norwegian Institute of Public Health, Zander Kaaesgate 7, Bergen, 5015 Norway; 4 Department of Teacher Education, NLA University College, Bergen, Norway; 5 Center for Alcohol and Drug Research (KORFOR), Stavanger University Hospital, Stavanger, Norway; 6 Center for Evaluation of Public Health Measures, Norwegian Institute of Public Health, Oslo, Norway

**Keywords:** Body weight perception, Body image, Body dissatisfaction, Trends, Social media, Adolescence, Children, Public health, Quality of life, Weight management

## Abstract

Increased exposure to social media and societal pressures to conform to idealized body standards may have amplified feelings of inadequacy among youth, notably in body weight perception. This study describes secular trends in body weight perception in Norwegian adolescents from 1994 to 2022, both before and during a period of technologically transformative changes proposed to influence body weight perception. To investigate trends in body weight perception, data across eight survey years from the Norwegian part of the repeated cross-sectional Health Behaviour in School-Aged Children study were used. Body weight perception was categorised as “Too thin”, “About right”, and “Too fat” among 11-, 13-, 15-, and 16-year-olds. The analyses showed stability in body weight perception across the 28-year period, with some gender and age differences observed. Small fluctuations were noted, most pronounced in the oldest age groups. The only significant change observed from 1994 to 2022, was an increase in the proportion of 16-year-old girls perceiving their body as “About right”. Between 2018 and 2022, there was an increase in the proportion of 11-year-old girls perceiving their body as “Too fat”. Stability in body weight perception was observed from 1994 to 2022, despite the assumed impact of certain technologically transformative changes, such as social media. However, the study highlights a concerning rise in 11-year-olds perceiving their bodies as “Too fat” between 2018 and 2022, underlining the continued need for regular monitoring of body weight perception in the adolescent population.

## Introduction

Body dissatisfaction is a phenomenon characterised by negative self-perceptions about physical bodily appearance such as weight or size^1^. These negative perceptions are a public health concern, given links to several negative psychological outcomes, including low self-esteem^2^, higher levels of stress^3^ poorer mental health^4,5^ as well as physical health^6^, and decreased life satisfaction^7^. Negative body weight perception (BWP) is particularly widespread among adolescents, with around half perceiving their bodies as either “Too thin” or “Too fat”^8^. Adolescence is characterised by a surge in hormonal activity, the onset of puberty, and significant physiological development mixed with psychological and emotion change^9^. This stage in life has been identified as critical for the onset and escalation of negative BWP, which often has antecedents in pre-adolescence and persists into adulthood^10^.

Adolescents’ body image and BWP are shaped by sociocultural, psychological, and biological factors. Media portrayals, peer comparisons, and family attitudes influence self-perception^11^, while psychological traits like self-esteem and resilience mediate these effects^12^. Biological changes during puberty may further increase body awareness, contributing to the multifaceted development of BWP. The Tripartite Influence Model (TIM) argues that media, family, and peers are the main influential factors impacting BWP^11^. According to the TIM, parents and peers can negatively influence adolescents’ BWP directly and indirectly through teasing, negative comments about weight, and demonstrating their own weight concerns^13^. These influences promote social comparisons and the internalization of societal values, which may lead to body dissatisfaction^11,14^.

Over the past decade, increased screen use and social media exposure, alongside societal pressures to conform to idealized body standards, may amplify feelings of inadequacy among youth. Studying these phenomena over time is crucial for identifying patterns, underlying causes, and the potential influence of emerging platforms or cultural shifts. Recent reports highlight a rise in negative BWP, particularly among adolescent girls^15^, though boys are also affected^16^. Social media, with its interactive content and easy accessibility, provides a potent platform for social comparison, a recognised predictor of body dissatisfaction^17^. The features of social media, combined with adolescent development factors as well as sociocultural emphasis on body-ideals have been argued to have created a “perfect storm” for exacerbating body image concerns, particularly in girls^18^. Meta-analyses show small to moderate links between social media use and negative BWP, particularly among girls^18,19^. As parental and peer support, factors reported to influence BWP^12,14^, declines in Europe, including Norway^20^, the growing influence of social media underscores the need to monitor BWP trends.

Trend studies on adolescent BWP prior to the turn of the millennia are scarce, and post-millennium research presents mixed results regarding changes in BWP, underscoring the complexity and variability of this issue over time. For instance, a Scottish study using data from the Health Behaviour in School-aged Children (HBSC) survey, which included 11, 13, and 15-year-olds from 1990 to 2014, found a consistent decrease over time in the number of girls perceiving themselves as “Too thin”^21^. Additionally, there was a decrease in the proportion of 11-year-old girls, and an increase in the proportion of 15-year-old boys who viewed themselves as “Too fat”^21^. Cross-national research based on the same large-scale study of mainly European countries indicated that the proportion of 15-year-old girls perceiving their bodies as “Too fat” remained relatively stable from 2002 to 2014 in 28 out of the 33 countries^16^. However, four countries saw an increase in the proportion with this perception, and one country showed a decline^16^. In contrast, the perception of being “Too fat” among boys increased in 10 countries during the same period, whilst 3 countries saw a decline, and the rest saw no changes^16^. Among American adolescents, studies also report stability in BWP over time. For instance, research on perceived overweight in adolescents from 1999 to 2007 showed few changes over time^22^, and a study on American college students also showed stability in body weight satisfaction from 1983 to 2007, except for a decrease amongst black women between 1990 and 1995^23^.

Studies have highlighted that social media, while being an influential platform for social comparison^17^, also offers diverse representations of body types. This diversity can help offset the pressure to conform to cultural body weight standards, contributing to stable BWP^24,25^. Norway, with the highest social media usage in the Nordic countries^26^ provides an interesting case study. With 90% of adolescents aged 9–18 using one or more social media platforms^26^, and 82% of the overall population being active social media users^27^, Norway’s early adoption of social media is driven by early widespread smartphone access^28,29^. Examining BWP in Norway can offer valuable insights into whether this high digital integration and social media usage has impacted adolescents’ perceptions of their body weight.

### Aims of the study

This study examines time trends in BWP in Norwegian adolescents from 1994 to 2022, a time before and during a period of technologically transformative changes proposed to influence BWP. These trends were explored across gender and age groups over the 28-year period.

## Methods

This study used data from eight survey years of the Norwegian part of the Health Behaviour in School-Aged Children study. The HBSC study is a cross-sectional survey conducted with four-year intervals in more than 50 countries in collaboration with the World Health Organisation^30^. The HBSC survey utilizes cluster sampling with school classes as the sampling unit to gather nationally representative student samples, and the survey is administered confidentially in classrooms using self-reported questionnaires. In Norway, a sample is collected from 11-, 13-, 15-, and 16-year-old students in primary and high school/upper secondary schools. Detailed information about the international study can be found elsewhere^30,31^. Overall, 48,345 adolescents responded to the questionnaires across the eight survey years, of which 2331(4.8%) were excluded from analysis due to missing data, leaving a total of 46,014 (49.8% girls) adolescents with valid data.

### Body weight perception

BWP, encompassing individuals’ subjective views on their body weight, was assessed with the adolescents being asked the question “What do you think about your body? I think it is..”, with answer categories of “Much too thin”, “A bit too thin”, “About the right size”, “A bit too fat”, and “Much too fat”. The single-item question has shown good validity and reliability and has been found to accurately capture adolescents’ self-perception of body size^32^.

In 1994, 1998, 2018 and 2022, a sixth answer category, “I don’t think about it” was included, which was reported by 15.4% across the four survey years. For the purpose of trend analysis, “About the right size” and “I don’t think about it” were collapsed into a single category (“About right”) representing an absence of perceiving one’s body negatively. Only 1.2% of the respondents perceived their body as “Much too thin” and 5.5% “Much too fat”, hence these categories were collapsed with ‘A bit too thin’ and ‘A bit too fat’, respectively.

### Gender and age

Gender was assessed with the question “Are you a boy or a girl?”. The age groups correspond to the students’ self-reported academic grade membership, namely the 6th (typically 11-year-olds), 8th (typically 13-year-olds), and 10th (typically 15-year-olds) grades, as well as the initial year of high school or upper secondary school (typically 16-year-olds).

### Statistical analysis

Data was analysed with IBM SPSS Statistics version 28.0.1.1. (15). The adolescents that had provided valid responses on gender, BWP, and age were included in the analyses. Chi-square tests of independence were conducted to examine the relationship between BWP (“Too thin”, “About right”, “Too fat”) and survey year (1994–2022) in the overall study population in girls and boys, and across the four age groups. As the chi-square test is sensitive to sample size^33^, statistical significance was set at *p* < .001 for analysis involving the total study sample and *p < *.05 when conducting tests between two survey years, and within groups.

## Results

In the total sample over the span of 28 years, most adolescents perceived their body to be “About the right size” (56.5%), followed by “Too fat” (30.0%) and “Too thin” (13.5%). The distribution of adolescents across BWP categories differed by gender (X^2^ (2, *N* = 46014) = 2080.42, *p* < .001), and age (X^2^ (6, *N* = 46014 = 1244.35, *p* < .001). The group with the highest proportion perceiving their body as “About right” was 11-year-old boys (72.7%). A larger proportion of girls (39.6%) compared to boys (20.6%) perceived their body to be “Too fat”. More boys (17.0%) than girls (10.1%) perceived their body as “Too thin” (Table 1).

**Table 1:** Sample characteristics, percentage of participants in each BWP category 1994–2022.

Age & Gender	*N*	1994	1998	2002	2006	2010	2014	2018	2022
N	46,014	6002	6761	6983	6250	5634	4187	5222	4975
“Too thin”									
11 boys	644	10.9	10	11.8	9.4	11	11.5	9.8	8.9
11 girls	655	11	11.1	13.1	9.2	11.6	9.5	7.4	11.3
13 boys	822	17.2	16.1	14.8	14	13.6	14.3	13.2	16
13 girls	600	13.5	12.4	11.4	9.4	9.4	10.1	9.4	11.9
15 boys	1031	21.1	19.7	19	19.6	15.5	22.3	19	23.4
15 girls	514	12.2	11.6	9.7	10.7	5.4	9.2	9	12.3
16 boys	1418	27.1	25.1	19.5	22.9	20.6	19.8	24.4	23
16 girls	542	11.2	11.5	10.1	7.5	7.1	6	7.8	8
“About right”									
11 boys	4503	72.7	71.4	71.6	72.5	71.8	71.8	77.1	72.8
11 girls	4056	62.5	64.5	61.8	66.5	63	69.2	72.5	59.7
13 boys	3439	60.7	63.7	64.4	63.5	63.7	63.9	64.2	61.5
13 girls	2728	48.4	49.2	53.6	49.1	51.7	54.7	50.5	46.9
15 boys	3038	58.3	60	60.8	57.6	57.5	52.7	58.2	56.5
15 girls	2167	42.6	36.8	47.9	39.6	43.6	42.9	44.5	46.9
16 boys	3451	51.5	56.1	61.4	58.4	50.6	52.9	53.8	53.2
16 girls	2591	37	40.4	44.2	41.5	39.7	37.5	45.3	45.9
“Too fat”									
11 boys	1044	16.4	18.6	16.6	18.1	17.2	16.7	13.1	18.2
11 girls	1526	26.5	24.4	25.1	24.3	25.4	21.3	20.1	29
13 boys	1188	22.1	20.2	20.8	22.5	22.7	21.8	22.6	22.6
13 girls	2080	38.1	38.4	35	41.5	38.9	35.2	40.1	41.2
15 boys	1158	20.6	20.3	20.2	22.8	27	25	22.8	20.1
15 girls	2381	45.2	51.6	42.4	49.7	51.1	47.9	46.5	40.8
16 boys	1361	21.4	18.8	19.1	18.7	28.7	27.3	21.8	23.8
16 girls	3077	51.7	48.1	45.6	51.1	53.2	56.5	46.8	46.2

Note: The average number of participants in each age group each year is 1438.

**Fig. 1:**
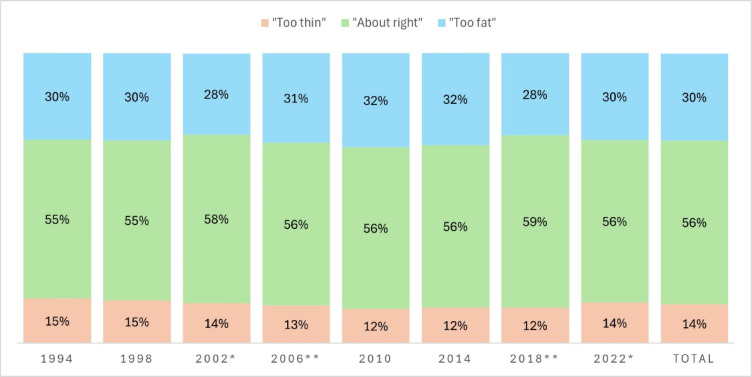
Distribution of adolescents perceiving their body as “Too thin” “About right” and “Too fat” over time (1994–2022). Note: Significant differences from previous year at * *p* < .05 level and ** at *p* < .001 level.

There were overall statistically significant differences in the proportion of adolescents in each BWP category across the 28-year period (X^2^ (14, *N* = 46014) = 97.04, *p* < .001), as well as differences between certain survey years (Fig. 1), however there were no obvious trends across time.

**Fig. 2:**
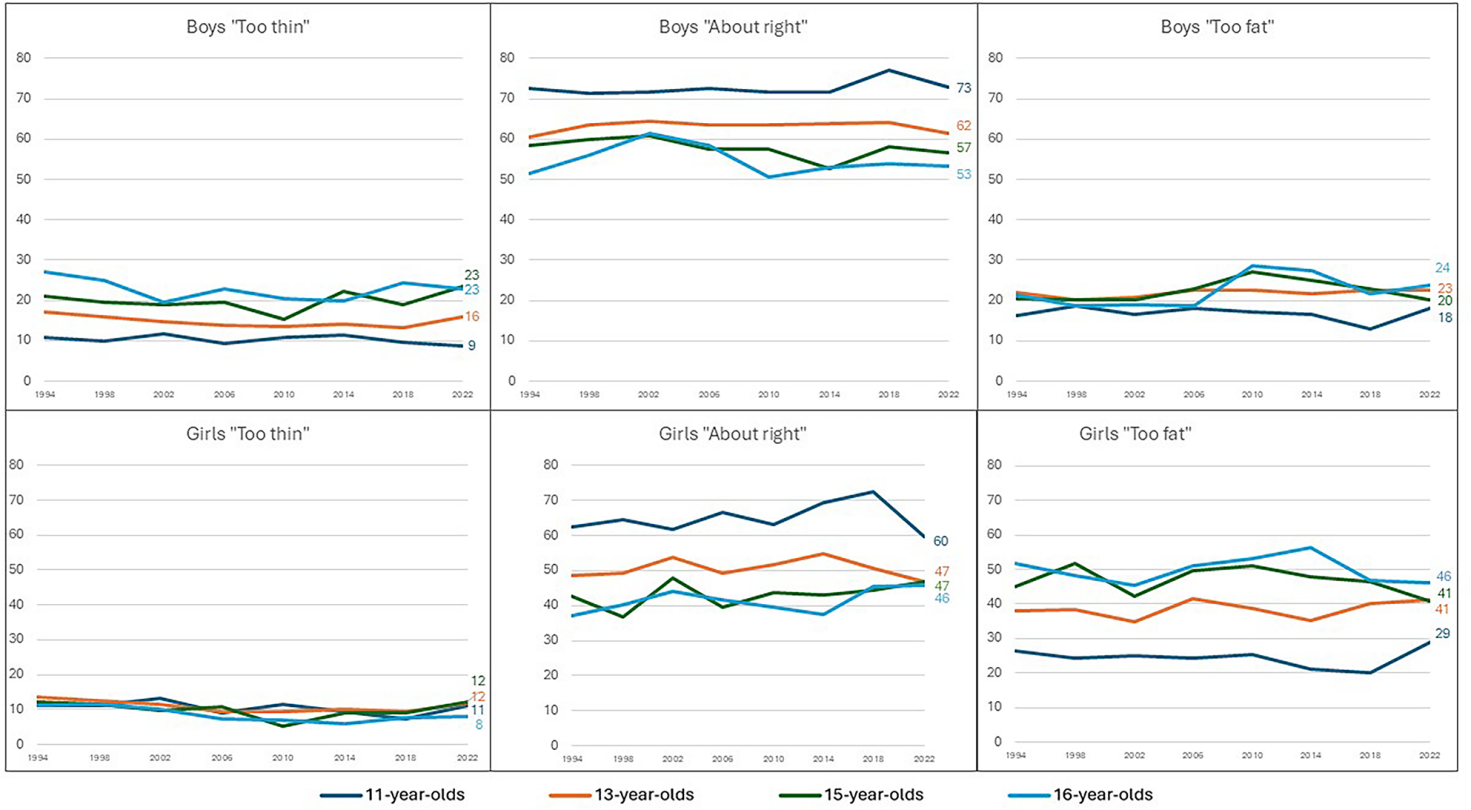
Distribution of BWP by gender and age over time (1994–2022).

Figure 2 display the proportion of boys and girls perceiving their body as “Too thin” “About right” and “Too fat” disaggregated by age across 8 survey years. No significant changes were observed in BWP categories when comparing the first and last survey years among boys. There were a few fluctuations across the years. Among 11-year-olds there was an increase in the proportion perceiving their body as “About right” between 2014 and 2018, followed by a non-significant decrease between 2018 and 2022. Among 15-year-olds, the proportion perceiving their body as “Too thin” decreased from 2006 to 2010 and increased in 2014. Among the 16-year-olds perceiving their body as “About right” decreased and “Too fat” increased between 2006 and 2010. Perceiving their body as “Too fat” then decreased between 2014 and 2018.

Among girls, more fluctuations were found in BWP categories over time (see Fig. 2). However, the only significant change in any BWP category between the first and last survey years was in 16-year-old girls perceiving their bodies as “About right”, with a larger proportion reporting this in 2022. In 11-year-olds, the proportion perceiving their body as “About right” increased from 2010 to 2014 followed by a decrease in 2022, reverting to levels similar to those observed prior to the increase. Among 15-year-olds perceiving their body as “About right”, an increase occurred between 1998 and 2002, followed by a decrease in 2006. In 16-year-olds, an increase was observed between 2014 and 2018.

The proportion perceiving their body as “Too thin” decreased among 11-year-olds between 2002 and 2006, followed by an increase between 2018 and 2022. Among all the other age groups the proportion remained stable apart from decreasing levels from 2006 to 2010 among the 15-year-olds, and from 2002 to 2006 among the 16-year-old girls. In addition, the perception of being “Too fat” increased distinctly from 2018 to 2022 in 11-year-olds. For 13-year-old girls, the proportion increased from 2002 to 2006. Among 15-year-old girls, there were significant fluctuations between 1994 and 2006, with lower levels in 2002. Among 16-year-old girls, the proportion increased from 2002 to 2006, followed by a decrease from 2014 to 2018.

## Discussion

This study provides an overview of secular trends in BWP among Norwegian adolescents aged 11–16 from 1994 to 2022. Overall, the results show minimal changes in the distribution of adolescents’ BWP over the 28-year period. In the stratified analysis, however, some variations were observed across age and gender. The only change between the first and last survey year was among 16-year-old girls, with an increase in the proportion perceiving their bodies as “About right”. Fluctuations were noted most frequently for the two oldest age groups. However, from 2018 to 2022, there was a marked increase in the number of 11-year-old girls perceiving themselves as “Too fat” as well as “Too thin”.

Our finding of stable body weight perceptions among adolescents aligns with Cash & colleague’s^23^ and other studies’^22^ overall findings among American youth from the mid-1990s to 2006, with the current study suggesting continuing stability in BWP. Minimal changes in BWP are also in general agreement with an international HBSC trend study in 33 countries conducted among 15-year-old girls from 2002 to 2014^16^.

The reason for the stability among Norwegian adolescents and an increase in 16-year-old girls perceiving themselves as “About right” remains unclear. Consistency in BWP over such a long period may suggest a “cultural norm” or “social reality” across time and space. According to TIM, peers, family and the media are the main factors influencing how individuals perceive their body weight^11^, and research shows that low social support correlates with negative body perception^12^. Interestingly, in Norway and other European countries, perceived social support from both peers and family has decreased in recent years^20^. Despite this, the expected increase in negative BWP was not observed in the current study. There was actually an increase over time in 16-year-old girls perceiving themselves as “About right”.

BWP is assumed to be a complex phenomenon influenced by several factors that interact. Another key factor influencing BWP, the media, has substantial change in the past decades, with the emergence of social media. Since its inception in the late 1990’s, social media use has increased globally^34,35^. In Norway in 2022, between 85 and 99% of 11–16-year-old use social media, particularly visually-oriented platforms^26^. These platforms, like Instagram, Snapchat, and TikTok, are linked to body image concerns^25^, dissatisfaction with specific body parts^36,37^ and dissatisfaction with weight and shape^38^. A study found that Norwegian adults are influenced by “healthism” and media-depicted body ideals^39^. Consequently, changing beauty standards on social media could influence beauty ideals, leading many to feel dissatisfied with their own bodies^40^.

A close look at social media could, however, contribute to some understanding of this stability. While social media can potentially negatively impact body image by promoting unattainable standards, it also offers arenas for support and community^41^, such as promoting body positivity and share experiences^42^, as well as advocating for greater acceptance of all body types, challenging traditional beauty standards^43^. Diverse representations of bodies and body-positive content on social media could help counterbalance the pressure to conform to a single standard, possible contributing to stable BWP among this age group^24,25^.

Furthermore, the overall stable time trends in BWP were observed in a period of increase in overweight and obesity among Norwegian adolescents between 1995 and 2011 follow by stable trends^44^. This is interesting as an association between body weight and body weight perception is well-documented, with higher weight status consistently linked to lower body satisfaction^45–47^. One possible explanation researchers have proposed is a “generational shift”, towards overweight being the new “normal” in today’s society^48,49^. Fewer adolescents living with overweight and obesity now perceive themselves as “Too fat” compared to the 1990s^50^ and early 2000s^51^. Also, a HBSC study from 2023 found that weight underestimation increased, whilst overestimation decreased between 2002 and 2018^52^. Visual normalization theory, a theory based on the idea that weight perception is judged relative to body size norms, argues that the commonality of larger body sizes in today’s society has shifted perceptions of what a “normal” weight body looks like, in turn raising the perception threshold for what is considered “Too fat”^53^. In addition, social comparison theory suggests that adolescents compare themselves to peers that are similar to them, rather than absolute standards^54^. These “misclassifications” may have contributed to the stable trends of BWP over the time period.

Nevertheless, the results show a concerning change in the recent pattern of 11-year-olds perceiving their bodies as “Too fat”, with an increase among girls between 2018 and 2022. According to a report on social media use among Norwegian adolescents, the proportion of children using social media increases rapidly between the ages of 10 and 11, and even more so between 11 and 12 years^55^. Recent studies have found that adolescents with higher media literacy are less likely to experience body dissatisfaction^56,57^. Given that 11-year-olds may have limited online media literacy as well as coping and regulatory skills compared to older adolescents, and are increasingly exposed to social media during this phase, they could be more vulnerable to its content, potentially impacting their BWP. The results reinforce the necessity to continue monitoring younger age groups in future studies to see whether the increase in negative BWP persists.

## Limitations and directions for future research

The present study is unique as it examined patterns in BWP of nationally representative samples of adolescents in Norway, spanning 28 years. Also, the application of a robust, standardised methodology throughout the survey years enhances the generalisability of the results. Limitations do however need to be acknowledged, such as the notion that the self-reported questionnaires possibly lead to response biases, like social desirability^58^. Further, the study measures BWP with a single item. Although the item has remained the same across all survey years in HBSC, thereby enabling trend analysis, it may be possible that a more comprehensive measure would capture a broader range of perceptions and nuances in body weight perception, such as assessing body area satisfaction, where adolescents evaluate each body part separately^23^. Moreover, in order to focus solely on trends in BWP, this study did deliberately not account for factors commonly linked to BWP, including BMI^59^ and other elements such as self-esteem and social support^12^. Incorporating these factors might reveal different trends in BWP.

To investigate the potential effects of social media and technological advancement use on BWP, there is a need for further trend studies in the coming years. In addition, studies are recommended to use measures that covers additional dimensions of body image, such as appreciation in addition to dissatisfaction with specific body parts to balance the focus away from solemnly a pathological approach to young peoples’ body perception. Future research is also recommended to investigate the interconnections among social media and body image more in depth, and the possible protective factor that media literacy may have on BWP. For instance, studies exploring how specific social media practices, such as upward social comparison and self-presentation, influence BWP are needed. These types of behaviours on social media have been associated with poor mental health, poor quality of life, and eating disturbances in adolescents^60–62^.

## Conclusion

In conclusion, overall stability of BWP among adolescents from 1994 to 2022 was observed, likely a result of a complex interplay of various factors. However, the concerning increase in the number of 11-year-old girls perceiving themselves as “Too fat” in 2022 underscores the need for continued regular monitoring of BWP, and also relational practices. Future research should focus on understanding the nuanced effects of social media on BWP, aiming to establish a better knowledge base to foster a healthier BWP among adolescents.

## Data Availability

The datasets supporting the conclusions of this article are available upon request from the HBSC Data Management Centre at The University of Bergen (https://www.uib.no/en/hbscdata). Access to the newest (2022) data is restricted to members of HBSC until its embargo is lifted in October 2026.

## Abbreviations

BWP: Body Weight Perception
HBSC: Health Behaviour in School-aged Children
BMI: Body Mass Index
TIM: Tripartite Influence Model
NSD: Norwegian Social Science Data Services
REK: Regional Research Ethics Committee
